# Pelvic floor muscle training and adjunctive therapies for the treatment of stress urinary incontinence in women: a systematic review

**DOI:** 10.1186/1472-6874-6-11

**Published:** 2006-06-28

**Authors:** Patricia B Neumann, Karen A Grimmer, Yamini Deenadayalan

**Affiliations:** 1 PhD candidate, School of Health Sciences, University of South Australia, Adelaide, Australia; 2 Director, Centre for Allied Health Evidence, University of South Australia, Adelaide, Australia; 3 Research Assistant, Centre for Allied Health Evidence, University of South Australia, Adelaide, Australia

## Abstract

**Background:**

Stress urinary incontinence (SUI) is a prevalent and costly condition which may be treated surgically or by physical therapy. The aim of this review was to systematically assess the literature and present the best available evidence for the efficacy and effectiveness of pelvic floor muscle training (PFMT) performed alone and together with adjunctive therapies (eg biofeedback, electrical stimulation, vaginal cones) for the treatment of female SUI.

**Methods:**

All major electronic sources of relevant information were systematically searched to identify peer-reviewed English language abstracts or papers published between 1995 and 2005. Randomised controlled trials (RCTs) and other study designs eg non-randomised trials, cohort studies, case series, were considered for this review in order to source all the available evidence relevant to clinical practice.

Studies of adult women with a urodynamic or clinical diagnosis of SUI were eligible for inclusion. Excluded were studies of women who were pregnant, immediately post-partum or with a diagnosis of mixed or urge incontinence. Studies with a PFMT protocol alone and in combination with adjunctive physical therapies were considered.

Two independent reviewers assessed the eligibility of each study, its level of evidence and the methodological quality. Due to the heterogeneity of study designs, the results are presented in narrative format.

**Results:**

Twenty four studies, including 17 RCTs and seven non-RCTs, met the inclusion criteria. The methodological quality of the studies varied but lower quality scores did not necessarily indicate studies from lower levels of evidence. This review found consistent evidence from a number of high quality RCTs that PFMT alone and in combination with adjunctive therapies is effective treatment for women with SUI with rates of 'cure' and 'cure/improvement' up to 73% and 97% respectively. The contribution of adjunctive therapies is unclear and there is limited evidence about treatment outcomes in primary care settings.

**Conclusion:**

There is strong evidence for the efficacy of physical therapy for the treatment for SUI in women but further high quality studies are needed to evaluate the optimal treatment programs and training protocols in subgroups of women and their effectiveness in clinical practice.

## Background

### Aim

The aim of this review was to critically appraise relevant peer-reviewed reports of original investigations of the efficacy or effectiveness of pelvic floor muscle training (PFMT) performed alone and together with other adjunctive physical therapies (eg biofeedback, electrical stimulation, vaginal cones) for stress urinary incontinence in women published in the last decade (1995–2005).

### Background and rationale

The International Continence Society defines urinary incontinence (UI) as the complaint of any involuntary leakage of urine [1]. It is a widespread [2] and prevalent condition affecting an estimated 1.8 million community-dwelling women over the age of 18 years in Australia [3]. The personal financial costs for women managing UI in Australia in 1998 were estimated at A$372 million per annum and the total annual costs of treatment at A$339 million [4].

Stress and urge incontinence are the two most common types of UI, which co-exist as mixed incontinence. Urine leakage is classified according to what is reported by the woman (symptoms), what is observed by a clinician (signs) and on the basis of urodynamic studies. Stress urinary incontinence (SUI) is the complaint of involuntary leakage on effort or exertion, sneezing or coughing (symptom) or the observation of urine leakage at the same time as the exertion (sign). SUI is the most common type of UI. Urge urinary incontinence (UUI) is the complaint of involuntary leakage accompanied or immediately preceded by, urgency [1]. Both are amenable to conservative therapy but surgery has conventionally been offered for SUI and medication with behavioural methods for UUI. The efficacy of surgery is variable [5-7]. Pharmacotherapy for SUI has also been developed but not extensively prescribed [8]. Since 1992, conservative management of UI has been promoted by the US Department of Health and Human Services (AHCPER) as first-line treatment for SUI for its efficacy, low cost and low risk [9].

SUI occurs when intra-vesical pressure exceeds urethral closure pressure in the absence of a detrusor contraction. SUI may be due to bladder neck hyper-mobility or poor urethral closure pressure [1]. The pelvic floor muscles (PFM) function to elevate the bladder, preventing descent of the bladder neck during rises in intra-abdominal pressure and to occlude the urethra. The theoretical basis for physical therapy to treat SUI is to improve PFM function by increasing strength, coordination, speed and endurance [10] in order to maintain an elevated position of bladder neck during raised intra-abdominal pressure with adequate urethral closure force [11].

A distinction is to be made between the terms 'efficacy' and 'effectiveness'. Efficacy is defined as "the probability of benefit to individuals in a defined population from a medical technology applied for a given medical problem under ideal conditions of use". By contrast, effectiveness is considered to have all the attributes of efficacy but to reflect "performance under ordinary conditions by the average practitioner for the typical patient" [12].

Pelvic floor muscle training (PFMT) and other physical therapies for the treatment of female SUI [13] and UI [14-16] has been the subject of previous systematic reviews. All of these reviews limited their inclusion criteria to randomized controlled trials, because this type of study design is considered to provide the best evidence of efficacy for an intervention by attempting to minimize biases and confounding variables [17].

Because of the very rigor of an RCT, it may not necessarily be appropriate to generalise the results of such a carefully controlled trial into clinical practice. Thus a treatment modality with demonstrated efficacy in an RCT may not be effective when combined with other modalities for a different patient population in clinical practice [12,18,19]. Subjects for RCTs are selected according to strict and often limited criteria, health personnel are highly trained and a standardized intervention is applied to all subjects, regardless of individual subject characteristics and clinical presentations (eg severity of incontinence, PFM function (strength, endurance, awareness)[20,21]. In clinical practice, physiotherapists are trained to provide treatment based on individual assessment and clinically reasoned processes, for patients presenting with incontinence and with a range of co-morbidities. Thus different treatment modalities (adjunctive therapies) may be applied to individual patients in conjunction with PFMT in order to activate a weak muscle, to improve sensory feedback, to enhance patient cooperation and compliance with an exercise program [22]. Observational studies provide the opportunity to establish the effectiveness of such interventions in routine clinical practice [19]. This is difficult to achieve in randomized trials [19] other than pragmatic randomized trials [23].

The effectiveness of physical therapy in clinical practice may thus be assessed from the evidence from lower level studies i.e. levels III & IV according to the Australian National Health and Medical Research Council's hierarchy of evidence [24]. These studies would be more likely to report on cohorts or case series of patients, treated under typical clinical conditions. In addition, such studies could also provide other information about clinical practice, such as the responsiveness to treatment (length of time taken to respond) not otherwise available from an RCT. No systematic review on SUI has reported on the generalisability (external validity) of the study findings and their applicability in clinical practice. External validity is an important aspect of methodological quality, but there are few critical review tools to evaluate whether the procedures, hospital characteristics and patient samples reported in the literature are relevant to clinical practice [25].

### Objective

This systematic literature review evaluated the evidence for the efficacy and effectiveness of physical therapy, described as pelvic floor muscle training with, and without, adjunctive physical therapies such as biofeedback, electrical stimulation or vaginal weights for the treatment of SUI in women.

The review addressed the following research questions:

1. What is the evidence for PFMT, either alone or in combination with adjunctive therapies, when considering all treatment protocols, for the treatment for SUI in women, in the short and medium terms (up to 12 months after treatment)?

2. What is the evidence for different types of PFMT?

3. What other reported factors could affect outcome of physical therapy?

4. What is the optimal period of treatment and number of treatments?

5. What is the effectiveness of physical therapy in clinical practice settings and can the findings in the research settings be generalised to clinical practice?

## Methods

### Criteria for inclusion in this review

The methods for conducting this systematic review and for assessing the quality of the evidence are based on the processes outlined by the Joanna Briggs Institute [26] and the Centre for Reviews and Dissemination at the University of York [21].

### Types of studies

In order to better understand whether those interventions which have demonstrated efficacy in the research setting are also effective when applied in the clinical setting, prospective research designs other than RCTs were also considered in this review. These included quasi-experimental, controlled clinical trials, observational studies and case studies/series. It was anticipated that these types of research designs may provide information about patient populations more typical of those encountered in primary care settings eg with a broad range of inclusion criteria. This information is needed to underpin estimates of the costs of treatment in the primary care setting.

In this review, experimental studies were classified as RCTs when randomly allocated intervention groups were compared, where a distinct control group could receive either another treatment modality or 'no treatment'. Thus studies were eligible for inclusion if there was at least one arm with a PFMT protocol, alone or together with other adjunctive therapies, compared with either a control group of 'no treatment' or 'usual treatment' or a different PFMT protocol, alone or together with other adjunctive therapies (biofeedback, electrical stimulation or vaginal weights).

Study designs without a control group but with a PFMT protocol, alone or together with other adjunctive therapies were also included. Studies or arms of studies which did not have a PFMT protocol and retrospective analyses or audits, which were unlikely to provide robust evidence of effectiveness because of time-based bias, were excluded.

Only peer-reviewed studies published in English in the last decade (1995–2005) were included in this review. The search was limited to the last decade in order to source the most recent, high-quality evidence [27]. This decision was justified on the grounds that systematic reviews evaluating the earlier literature found many of the included studies to be of poor or moderate methodological quality [13-15] and based on the findings of Moseley et al (2002), it was assumed that the more recent literature was more likely to be of higher methodolgical quality.

### Types of participants

The study populations considered in this review included subjects who were adult females of any age, not pregnant or within six weeks post-partum, with a clinical or urodynamic diagnosis of SUI. Clinical diagnosis could be based on the self-report (symptom) and/or sign of stress incontinence. Studies were excluded if they included subjects with mixed UI or detrusor overactivity because of the assumption of a different underlying pathology and thus rationale of treatment, even if outcomes for subgroups of women with SUI were reported.

### Types of interventions

#### Inclusions

Any PFMT i.e. pelvic floor muscle exercises, with application of a specific training protocol or PFMT together with any combination of adjunctive therapies: biofeedback (BF), electrical stimulation (ES), vaginal weights or cones (VW). All types of BF were included if it was used to enhance the awareness of a correct PFM contraction: EMG (electromyography, either vaginal or surface abdominal), vaginal squeeze pressure or ultrasound. Biofeedback could be used to enhance teaching of the correct response or to train repetitive PFM contractions.

ES included any low or medium frequency current applied externally (interferential currents) or internally via a vaginal electrode.

#### Exclusions

Interventions that included any of the therapies listed above as adjunctive, either alone or in combination, without a PFMT protocol. Thus in studies which included a subgroup which was treated with one or more adjunctive therapies without a specific PFMT protocol, the results of the subgroup were excluded from the analysis. Thus BF, ES and VW were not considered on their own or together unless they were part of program with a PFMT protocol. Adjunctive therapies have been the subject of previous reports [15,28].

### Types of outcome measures

Only outcome measures relevant for clinical practice were reported in this review, thus urodynamic study measures were excluded.

The principal measures of effectiveness were considered to be the proportion of women cured (continent/dry), and the proportion of women whose symptoms were improved based on clinical measures such as pad tests, urinary diaries or quality of life scores.

In line with the recommendations of the International Continence Society, outcomes were considered the under the following five categories [29]:

#### A. Women's observations (subjective measures)

• Perception of cure and improvement

#### B. Quantification of symptoms (objective measures)

• Pad changes over 24 hours (self-reported)

• Incontinent episodes over 24 hours (self-completed bladder chart)

• Pad tests of quantified leakage (mean volume or weight of urine loss)

#### C. Clinician's observations

• Objective assessment of pelvic floor muscle strength

#### D. Quality of life

• General health status measures (physical, psychological, other)

• Condition-specific health measures (specific instruments designed to assess incontinence)

#### E. Socioeconomic measures

• Health economic measures

This review also included other information about progression to surgical intervention and adverse events. All outcome measures were documented and categorized under the headings described above.

### Search strategy

To identify all relevant studies for the review, the search strategy comprised searches of the following:

Bibliographic Databases:MEDLINE, CINAHL, AMED, Current Contents, The Cochrane Library, Cochrane Database of Systematic Reviews (CDSR), The Cochrane Controlled Trials Registers (CCTR), SPORTdiscus, CatchWord, AUSTHealth, Academic search elite, Science Direct, PubMed, Ageline, PEDro, OVID

Internet source: , 

Reference lists of systematic reviews, meta-analyses, reviews and the studies identified by the search strategy above were pearled for additional relevant source material. Their inclusion was validated by checking their key words against the search terms. Hand searching for published and unpublished data was not performed because a systematic and thus reproducible approach could not be guaranteed.

All relevant studies with an English language abstract were located for assessment against the inclusion criteria. Date of the last search was 20 May 2005. Individual strategies were developed for each source searched to accommodate search engine idiosyncrasies. The core terms and search strategies used for each literature source are listed in additional file 1.

### Eligibility criteria

#### Study selection

Relevant articles were identified from the hits produced from each library database, internet source or reference lists by applying the eligibility criteria. The relevant eligible studies were documented in a Microsoft Excel (2000) database [see additional file 2].

The full text version of all relevant peer-reviewed studies was obtained where possible, and abstracts were only included as a proxy for the complete text if sufficient data was available in the abstract to assess and fulfil all the eligibility criteria, to critically appraise and to provide point measures on at least one measure of outcome. Inclusion of studies into this review was reached by consensus between the two reviewers.

### Assessment of methodological quality

#### Level of evidence

The level of evidence of each retrieved study was assessed using the Australian National Health & Medical Research Council [24] levels of evidence [see additional file 3] in order to describe potential for bias.

#### Methodological quality

To evaluate the methodological quality of the included studies, each study was critically appraised by two independent reviewers using a purpose-built critical review instrument [see additional files 4 &5]. The purpose-built instrument was a modification of the tool developed by the McMaster University Occupational Therapy Evidence-Based Practice Research Group [30]. This appraisal tool is a critical review form for quantitative studies considering eight main points: study purpose, literature, study design, sample, outcomes, intervention, results, conclusions and clinical implications. Although this tool was designed for all types of quantitative studies, other authors have recommended a separate tool for each of the two main types of design: experimental and observational studies [31]. We developed our tools drawing on information from the Agency for Healthcare Research and Quality report 'Systems to Rate the Strength of Scientific Evidence' [31] and from the Centre for Reviews and Dissemination, University of York [21]. The modified tool developed for this review provides a maximum quality rating score of 23 for RCTs and a maximum score of 19 for non-RCTs. It was pilot-tested and modified a number of times before implementation to ensure content and face validity, and agreement on its application by the reviewers involved in this review. The final version of the purpose-built instrument was then applied by two reviewers working independently. They then compared critical appraisal scores and resolved disagreements in scoring by discussion.

Details of the quality assessment are provided [see additional files 4 &5] with studies ranked according to their quality assessment score to provide readers with an overview of their methodological quality. All the studies were then considered for the strength of their evidence, based on the quality score and with particular consideration of the factors which were concerned with control of bias. Studies with a high quality score were considered to show evidence of good control of bias (eg attention to random allocation processes, baseline similarity of groups, reliable outcome measures) as well as other factors concerning quality reporting, such as consideration of ethical processes and relevance of the literature review. Studies with a high quality score are identified and highlighted by the reviewers in the text for their contribution to evidence about treatment outcomes.

#### Data extraction

Relevant data was extracted from each study in a separate extraction sheet, providing a profile of each study using the following headings:

• Information about service delivery (health professional and setting/institution)

• Demographic information about the subjects in the study

• Study methods

• Descriptions of the intervention(s)

• Description of the outcome measure(s)

• Key results from data analysis – short term and at 12 months

Similar to the process of critical appraisal, both reviewers extracted information independently and where there was disagreement, consensus was reached by discussion or in consultation with a third party

#### Data synthesis

Because our review included studies of evidence levels II, III and IV (NHMRC 1999), and because study measures were not homogenous, it was not possible to analyse the data by meta-analysis. Thus findings are presented as narrative summaries. In studies with a 'no treatment' or 'usual treatment' control group, analysis of between-group effects were reported in this analysis. In studies without a control group, within-group changes were used to calculate treatment effects. All relevant outcomes ie those fitting the inclusion criteria, were reported, including statistically significant and non-significant findings.

## Results

### Methodological quality and description of studies

The search identified 7760 potentially relevant research reports in the period 1995–2005, of which 24 studies fulfilled the inclusion criteria and hence were considered in this review. Twenty one included studies were English peer-reviewed research reports, three were peer-reviewed conference abstracts with no published full-text report and one was a peer-reviewed foreign language paper with an English language abstract. This English abstract was used for data extraction. There was 100% agreement between the reviewers in terms of study inclusion. Summaries of the studies included in the review are provided in Tables 1 and 2. Studies are presented in order of their quality assessment score with information about the level of evidence, interventions investigated and information to determine the generalisability of the study findings.

**Table 1:** Summary of all studies with interventions, level of evidence, quality rating score and age

Studies	Intervention	Hierarchy of Evidence ^a^	Quality Rating Score (%)	Mean age (SD)^b^
Bo (1999)	PFMT v BF v ES v control	II	23/23 (100)	49.6 (10)
Morkved (2002)	PFMT v PFMT+BF	II	22/23 (96)	47.8 (8.2)
Dumoulin (2004)	PFMT+ES+BF v PFMT+ES+BF+Ab Ex v control	II	21/23 (91)	36.2 (median) (IQ range 23–39)
Bo (2000)	PFMT	II	21/23 (91)	49.6 (10)
Berghmans (1996)	PFMT v PFMT+BF	II	20/23 (87)	48 (range 18–70)
Knight (1998)	PFMT+BF v PFMT+BF+ES('home') v PFMT+BF+ES('clinic')	II	17/23 (74)	NR (range 24–68)
Miller (1998b)	PFMT (motor learning)	II	17/23 (74)	68.4 (range 60–84)
Parkkinen (2004)	PFMT+ES+BF+VW v PFMT+VW	III-2	14/19 (74)	46.8 (range 32–65)
Wong (2001)	PFMT+BF v PFMT+BF+Ab BF	II	16/23 (70)	46 (range 30–62)
Dumoulin (1995)	PFMT+ES+BF	IV	13/19 (68)	32 (9.5)
Johnson (2001)	PFMT (SVC) v PFMT (NMVC)	II	15/23 (65)	50 (35–65)
Hay-Smith (2002) A	PFMT (motor learning/strength) v PFMT (motor learning)	II	15/23 (65)	48.8 (13.2 SD)
Arvonen (2001)	PFMT v PFMT+VW	II	15/23 (65)	48 (range 28–65)
Cammu & van Nylen (1998)	PFMT+BF v VW	II	15/23 (65)	55.9 (9.5)
Turkan (2005)	PFMT+ES	III-2	11/19 (58)	47.6 (8)
Pieber (1995)	PFMT+BF v PFMT+BF+VW	II	13/23 (57)	43 (+/- 6)
Chen (1999)	PFMT+ES	IV	11/19 (58)	NS (range 20 to >50)
Glavind (1996)	PFMT v PFMT+BF	II	13/23 (57)	45 (median)(range 40–48)
Pages (2001)	PFMT v BF	II	13/23 (57)	51.1 (range 27–80)
Bidmead (2002) A	PFMT v PFMT+ES v PFMT+sham ES v control	II	10/23 (43)	NR
Sung (2000)	PFMT	III-2	8/19 (42)	range 18 – >60
Aksac (2003)	PFMT v PFMT+BF v control	II	9/23 (39)	52.9 (7.2)
Balmforth (2004) A	PFMT+BF	IV	6/19 (32)	49.5 (10.6)
Finkenhagen (1998) A	PFMT	IV	5/19 (26)	49 (range 25–67)

A = available in English only as abstract; ^a ^= According to Australian National Health and Medical Research Council Hierarchy of Evidence (1998); ^b ^= Mean age (SD) unless otherwise stated; PFMT = pelvic floor muscle training; ES = electrical stimulation; BF = biofeedback; VW = vaginal weights; PT = physiotherapist; UDS = urodynamics studies; NR = not reported, SVC = submaximal voluntary contraction, NMVC = near-maximal voluntary contraction

**Table 2:** Summary of studies with factors pertaining to external validity

Studies	Diagnosis	Intervention by	Setting	Excluded if prior surgery	Volunteers (V) or Referred (R)
Bo (1999)	S, Pad T, UDS	PT	Multicentre	yes	V+R
Morkved (2002)	S, Pad T, UDS	PT	NR	yes	V
Dumoulin (2004)	S, Pad T, UDS	PT	NR	yes	V
Bo (2000)	S, Pad T, UDS	PT	NR	yes	NR
Berghmans (1996)	S, CST, Pad T, UDS	PT	PT clinic	yes	R
Knight (1998)	UDS	PT	Tertiary Clinic	no	NR
Miller (1998)	S, CST	NR	NR	yes	NR
Parkkinen (2004)	S, Pad T, UDS	PT	Hospital PT clinic	no	NR
Wong (2001)	S, UDS	PT	Hospital PT clinic	yes	R
Dumoulin (1995)	S, Pad T, UDS	PT	NR	NR	V
Johnson (2001)	S, UDS	NR	NR	yes	V+R
Hay-Smith (2002)	S, CST, Pad T	PT	NR	yes	V+R
Arvonen (2001)	S	PT	OP PT clinic	no	R
Cammu & van Nylen (1998)	S, UDS	PT	NR	no	NR
Turkan (2005)	S, Pad T, UDS	PT	University PT clinic	yes	R
Pieber (1995)	UDS	PT	Urodynamic unit	yes	R
Chen (1999)	S, CST, Pad T, UDS	NR	NR	yes	R
Glavind (1996)	S, Pad T, UDS	NR	NR	yes	NR
Pages (2001)	S, UDS	PT	OP hospital clinic	no	R
Bidmead (2002)	UDS	PT	NR	NR	NR
Sung (2000)	S	PT	NR	NR	R
Aksac (2003)	UDS	Therapist	NR	NR	NR
Balmforth (2004)	S, UDS	PT	NR	yes	R
Finkenhagen (1998)	NR	PT	PT clinic (primary care)	NR	NR

S = symptoms, Pad T = pad test, CST = cough stress test, UDS = urodynamic studies, NR = not reported, PT = physiotherapist, OP = outpatient

Arms of studies were excluded where there was no description of a specific PFMT protocol. Thus the following arm(s) were excluded: Cammu & van Nylen (1997) [32] (VW only), Sung et al (2000) [33] (ES/BF) and Bo et al (1999) [34](ES, VW).

#### • Hierarchy of evidence

There was initially 91% agreement (Cohen's Kappa: 0.8) between the reviewers regarding the level of evidence assigned to each study (NHMRC, 1999). A Kappa score of more than 80% is considered to represent 'excellent' agreement and between 60–80% 'substantial' agreement [35]. Complete agreement was reached after discussion.

Seventeen of the 24 studies identified were RCTs [32,34,36-50]. Seven were non-RCTs, of which three were level III-2 studies ie cohort or interrupted time series with a control group [33,51,52] and four were level IV studies ie case-series (before-after investigations) without a control group [53-56].

#### • Methodological quality of included studies

There was initially 83% agreement (Cohen's Kappa: 0.65) between the reviewers regarding the methodological quality of the included studies. After consultation, 100% agreement was reached. The methodological quality of the studies was variable with the highest scoring 100% (23/23) [34] and the lowest (26%) 5/19 [55]. There was no correlation between a more recent date of publication and quality score (Pearson's correlation – 0.03, p > 0.05).

A summary of the quality assessment of the 17 level II studies [see additional file 4] and the seven level III & IV studies [see additional file 5] is provided. The methodological quality of the RCTs varied from 23/23 (100%) [34] to 9/23 (39%) [36]. The methodological quality of the level III and IV studies was also variable with scores from 14/19 (74%) [51] to 5/19 (26%) [55]. Studies with a lower quality score contained a number of sources of bias which should be considered when interpreting the results. However, the four studies in abstract form had limited information for quality assessment contributing to their lower quality scores.

### Types of participants

Women were included with a urodynamic diagnosis of SUI, a clinical diagnosis based on signs and/or symptoms, or a combination of the above [1]. There was considerable variation in the hormonal status and age (18–84 years) of subjects in this review. Two studies [41,56] specifically recruited younger, pre-menopausal women with SUI persisting at least 3 months after the last childbirth. These authors stated that this time was chosen to allow the hormonal changes from pregnancy and parturition to have resolved. Another study [49] also specifically recruited pre-menopausal women. By contrast, Miller et al (1998) recruited older women with a mean age of 68 (range 60–84) and Aksac et al (2003) reported on women with a mean age of 53 (SD 7.2) years who were all using oral hormone replacement therapy. All other studies investigated various combinations of PFMT and adjunctive therapies in women with a mean age 46–56 (range of 18–80). Some of these studies stated that their populations included women who were both pre- and post-menopausal [33,34,38,43,47,54]. There was therefore considerable heterogeneity in the studies reviewed in terms of possible confounding due to age and hormonal status.

### Identification and/or control of potential confounders

The following confounding variables were controlled by stratification in a number of studies: severity of symptoms [34,38,41,47], referral source [34,38,41,47] and parity [34].

The initial severity of incontinence was not always reported and methods used to describe severity varied considerably so that any comparisons should be made with caution (Table 3). Two studies included women with a past history of surgery for incontinence [45,51]. In twelve studies, it was stated that women were excluded if they had prior surgery for incontinence [34,38,41-43,46,47,49,50,52-54] and it was not reported in nine other studies [32,33,36,37,39,40,44,48,56].

**Table 3:** Baseline severity of symptoms: incontinent episodes (IE) and urine loss (g) (pad test)

Study	IE/day	IE/week	Urine loss (g) (pad test)
Aksac (2003)			20 (1 hour)
Arvonen (2001)			25 (SPT, st.b.vl)
Balmforth (2004)			12.2 (SPT, st.b.vl)
Berghmans (1996)	2–3		28 (48 hr pad test)
Bidmead (2002)			10 (SPT)
Bo (1999)	2.0 per 3 days		38.6 (SPT, st.b.vl.); 14.5 (24 hr pad test)
Bo (2000)			45 (SPT, st.b.vl)
Cammu & van Nylen (1998)		14.4	NR
Chen (1999)	5.5		20 (1 hour)
Dumoulin (1995)			74.4 (SD 84.3) (SPT, st.b.vl)
Dumoulin (2004)			PF group: 12.5 g: PF+ abs group: 20 g (SPT, st.b.vl)
Finkenhagen (1998)			NR
Glavind (1996)			10.9 (SPT, st.b.vl)
Hay-Smith (2002)	1.8		3.9 ml (paper towel test)
Johnson (2001)	3.6 (range: 1.86–13)		12.9 (range: 1.76–111.42) (10 hour pad test)
Knight (1998)			14.6 (SPT, st.b.vl)
Miller (1998)			Paper towel test
Morkved (2002)			27.5 (SPT, st.b.vl), 42.2 (48 hr pad test)
Pages (2001)			NR
Parkkinen (2004)			(SPT, st.b.vl)
Pieber (1995)			NR
Sung (2000)			NR
Turkan (2005)			(1) 8.6 (2) 29.1 (3) 236.4) (1 hour pad test)
Wong (2001)		6.3	10.8 (SPT, standardised fluid intake)

SPT = stress pad test; st.b.vl = standardised bladder volume

Recruitment methods varied across the included publications, which potentially influenced subjects' responses to intervention. In three studies, the participants were volunteers who responded to newspaper advertisements [47] or from outpatient hospital populations [41,56]. In three studies, participants were both volunteers and referred [34,43,44]. In ten other studies, they were referred by a medical practitioner or recruited from a tertiary institution clinic population [33,37,38,45,48-50,52-54] and in the remaining studies the source was not reported [32,36,39,40,42,46,51,55].

### Types of interventions

The studies were divided into intervention categories and results summarised according to the different interventions reported: 14 studies reported on PFMT alone (Table 4), 11 studies on PFMT with BF (Table 5), three studies on PFMT and ES (Table 6), two studies on PFMT and VW (Table 7), three studies on PFMT with BF and ES (Table 8), one study on PFMT, BF and VW, (Table 9), and one study on PFMT combined with ES, BF and VW (Table 10). Details of the protocols for the interventions for all studies are detailed in Table 11.

**Table 4:** Outcomes of studies of PFMT with percentage cure, cure/improvement and positive and statistically significant outcomes

PFMT studies	Treatment time	N (subjects)	N (% lost to follow-up)	% cure	% cure/improved	N (%) positive & statistically significant outcomes
Bo (1999)	6 months	29	4 (14)	44 (1), 56 (4)	48 (4)	8/9 (89)
Morkved (2002)	6 months	50	4 (17)	46 (1), 30 (4) 57 (2)	93 (4)	6 (100)
Bo (2000)	6 months	24	4 (8)	6–44 (5)	NR	1 (100)
Berghmans (1996)	4 weeks	20	0 (0)	15 (2)	85 (2)	1 (100)
Miller (1998b)	1 week	27	0 (0)	23 (3)	NR	2 (100)
Hay-Smith (2002)^a^	20 weeks	64	2 (3)	7 (4)	47 (4)	NR
Hay-Smith (2002)^b^	20 weeks	64	3 (5)	2 (4)	41 (4)	NR
Arvonen (2001)	4 months	20	1 (5)	26 (1)	58 (4)	3 (100)
Glavind (1996)	NR (2–3 sessions)	20	5 (25)	20 (1)	NR	NR
Pages (2001)	3 months	27	0 (0)	69 (4)	100 (4)	3 (100)
Bidmead (2002)	14 weeks	40	NR	NR	NR	3 (100)
Sung (2000)	6 weeks	30	NR	NR	NR	3 (100)
Aksac (2003)	8 weeks	20	NR	75 (3)	100 (3)	10 (100)
Finkenhagen (1998)	6 months	38	2 (5)	35 (4)	71 (4)	NR

Hay-Smith ^a ^= motor learning protocol, Hay-Smith ^b ^= strength and motor learning protocol NR = not reported; (1) = pad test with standardised bladder volume; (2) = 48 hour pad test; (3) = other types of pad test; (4) = self-rated assessment of incontinence; (5) = self-reported quality of life/sexual function domains

**Table 5:** Outcomes of studies of PFMT and BF with percentage cure, cure/improvement and positive and statistically significant outcomes

PFMT+BF studies	Treatment time	N (subjects)	N (% lost to follow-up)	% cure	% cure/improved	N (%) positive & statistically significant outcomes
Morkved (2002)	6 months	53	5 (9)	58 (1); 65 (2) 40 (3)	97 (3)	6 (100)
Berghmans (1996)	4 weeks	20	0 (0)	25 (2)	95 (2)	1 (100)
Knight (1998)	6 months	21	3 (14)	NR	72 (1) 56 (3)	2 (100)
Wong (2001) ^a^	4 weeks	19	0 (0)	NR	NR	3/5 (60)
Wong (2001)^b^	4 weeks	19	0 (0)	NR	NR	4/5 (80)
Johnson (2001) ^a^	6 weeks	16	0 (0)	25 (3)	NR	4 (100)
Johnson (2001) ^b^	6 weeks	16	0 (0)	38 (3)	NR	2/4 (80)
Cammu & van Nylen (1998)	12 weeks	30	0 (0)	53 (3)	NR	NR
Pieber (1995)	3 months	25	11 (44)	22 (3)	86(4)	NR
Glavind (1996)	4 weeks	20	1 (5)	58 (4)	NR	NR
Pages (2001)	1 month	24	11 (46)	62 (3)	100 (3)	1 (100)
Aksac (2003)	8 weeks	20	NR	80 (4)	100 (4)	8 (100)
Balmforth (2004)	14 weeks	97	NR	NR	NR	5 (100)

NR = not reported; (1) = stress pad test with standardized bladder volume; (2) = 48 hour pad test; (3) = self-report; (4) = 1 hour pad test; Wong ^a ^= vaginal BF; Wong ^b ^= vaginal BF plus rectus abdominis BF; Johnson ^a ^= Training with Submaximal Voluntary Contractions; Johnson ^b ^= Training with Near Maximal Voluntary Contractions

**Table 6:** Outcomes of studies of PFMT and ES with percentage cure, cure/improvement and positive and statistically significant outcomes

PFMT+ES studies	Treatment time	N (subjects)	N (% lost to follow-up)	% cure	% cure/improved	N (%) positive & statistically significant outcomes
Turkan (2005)	5 weeks	17	0 (0)	Total: 38 (1) a: 88; b: 1; c: 0	NR	4 (100)
Chen (1999)	3 months intensive, 21 m home training	72	0 (0)	7 (2)	61 (2)	NR
Bidmead (2004)	14 weeks	97	NR	NR	NR	3 (100)

NR = not reported; (1) = self-rated assessment of incontinence; (2) = other type of pad test; Turkan: % subjects cured in groups a,b,c stratified by baseline severity of incontinence based on 1 hour pad test a: mild incontinence: 0–2 g; b: moderate incontinence: >2–10 g; c: severe: >10 g

**Table 7:** Outcomes of studies of PFMT and VW with percentage cure, cure/improvement and positive and statistically significant outcomes

PFMT+VW studies	Treatment time	N (subjects)	N (% lost to follow-up)	% cure	% cure/improved	N (%) positive & statistically significant outcomes
Parkkinen (2004)	12 months	19	3 (16)	NR ^a^	NR ^a^	3 (100)
Arvonen (2001)	4 months	20	2 (10)	50 (1); 22 (2)	61 (2)	2 (100)

(1) = objective cure based on pad test with standardised bladder volume, (2) = subjective rating of cure; ^a ^= not reported at 12 months

**Table 8:** Outcomes of studies of PFMT, ES and BF with percentage cure, cure/improvement and positive and statistically significant outcomes

PFMT+ES+BF studies	Treatment time	N (subjects)	N (% lost to follow-up)	% cure	% cure/improved	N (%) positive & statistically significant outcomes
Dumoulin (2004) ^a^	8 weeks	21	1 (5)	70 (1)	90 (1)	8/9 (89)
Dumoulin (2004) ^b^	8 weeks	23	0 (0)	73 (1)	90 (1)	8/9 (89)
Knight (1998) ^a^	6 months	25	6 (24)	NR	53 (1) 47 (2)	2 (100)
Knight (1998) ^b^	6 months	24	4 (17)	NR	80 (1) 80 (2)	2 (100)
Dumoulin (1995)	3 weeks	10	2 (20)	62.5 (1)	100 (1)	3 (100)

(1) = Pad test with standardised bladder volumes, (2) = subjective report; Dumoulin ^a^: training protocol with PFMT, ES, BF; Dumoulin ^b^: training protocol with PFMT, ES, BF and specific deep abdominal muscle training; Knight ^a^: training protocol with PFMT, BF, ES ('home' low intensity, 10 Hz); Knight ^b^: training protocol with PFMT, BF, ES ('clinic' high intensity, 35 Hz)

**Table 9:** Outcomes of studies of PFMT, BF and VW with percentage cure, cure/improvement and positive and statistically significant outcomes

PFMT+BF+VW studies	Treatment time	N (subjects)	N (% lost to follow-up)	% cure	% cure/improved	N (%) positive & statistically significant outcomes
Pieber (1995)	3 months	21	8 (38)	38.5 (1)	84.5 (1)	NR

(1) = subjective rate of cure

**Table 10:** Outcomes of studies of PFMT, BF, ES and VW with percentage cure, cure/improvement and positive and statistically significant outcomes

PFMT+BF+ES+VW studies	Treatment time	N (subjects)	N (% lost to follow-up)	% cure	% cure/improved	N (%) positive & statistically significant outcomes
Parkkinen (2004)	12 months	19	2 (11)	NR ^a^	NR ^a^	3 (100)

NR ^a ^= not reported at 12 months

**Table 11:** Summary of interventions

Studies/arms of studies	Control group protocol	PFM action taught 1 = digital vaginal 2 = other	PFMT protocol (s/s) = Contraction time in seconds/relaxation time in seconds	Intensity of contract-ions or type of PFM T	Adjunct-ive therapy	Adjunctive therapy protocol	Duration of inter-vention	N of treat-ments (individual unless other-wise stated)
Aksac 2003 PFMT		1	5s/10s, 10 reps, 3 sets/day. After 2 weeks, 10s/20s relax. Weekly individual sessions.	NR. Relaxation of abdominals, gluteals			8 weeks	8

Aksac 2003 PFMT+BF		2	10s/20s, 40 reps, 3 sets/week. Weekly individual sessions.	NR	EMG vaginal BF to learn action only	No home training with BF.	8 weeks	8

Aksac 2003 Control group	No PFMT						NA	

Arvonon 2001 PFMT	NA	1	5s/5s, 10 reps (max), seated/standing, 2 sets/day. 3s/3s, 15 reps, (submax) 1 set/day 2 min. sustained (submax) 1 rep, 1 set/day. 3 clinic visits	Maximal, submaximal			4 months	3

Arvonon 2001 PFMT+VW	NA	1	a/a		VW	VW (50 g, 65 g, 80, 100 g) 20s/20s, (max) 10 reps, standing, 2 sets/day. 15 mins. VW with daily activities, gymnastics.	4 months	3

Balmforth 2004 PFMT+BF	NA	2 Perineal ultrasound	Intensive + individualised PFMT + 'behavioural modification' program Home program: NR	NR	Perineal ultrasound to teach correct contraction. Pre-treatment only.		14 weeks	NR

Berghmans 1996 PFMT	NA	1	3–30s contractions, 10–30 reps, supine/standing/all fours. PFE with coughing, stairs, lifting, jumping. Home: 3x/day.	NR			4 weeks	12

Berghmans 1996 PFMT+BF	NA	1	a/a	NR	EMG vaginal BF Clinic only.	Individual program for 12 sessions.	4 weeks	12

Bidmead 2002 PFMT		NR	'Conventional 'PFE by experienced research physiotherapist. Home: details NR	NR			14 weeks	NR

Bidmead 2002 PFMT+ES		NR			ES (no details reported)	Same PFE program with home ES	14 weeks	NR

Bidmead 2002 Control group	No treatment							

Bo 1999 PFMT		1	6–8s/6 s, 8–12 reps, 3–4 fast contractions at end of 'hold', 3 sets/day. Weekly group sessions with ex in different positions and for abdominals, back, thighs. Monthly PFM assessment.	High intensity			6 months	24 group 6 individual

Bo 1999 Control group	No contact. Offered Continence Guard							

Bo 2000 PFMT		NR	As for Bo 1999				6 months	24 group 6 individual

Bo 2000 Control group	No contact Offered use of Continence Guard							

Cammu & van Nylen 1998 PFMT+BF	NA	1	'Brief' + 10s contractions, 10 reps, as many sets as possible 'within patients capacity'. Home: Increasing number of sets	Maximal	BF vag EMG + 'abdominal' EMG to reduce Valsalva efforts	Individual: Weekly, 30 min BF session	12 weeks	6

Chen 1999 PFMT+ES	NA	1	No details. 15 mins 2 sets/day, 3 months Then 15 mins/day, 1 set/day, 21 months		ES intravaginal, home stimulator	Increasing tx times: 20,40, 60 min, 2/week, 3 months. Biphasic square wave, 25 Hz.	3 months (ES) 24 months (PFE)	24 + 6

Dumoulin 2004 PFMT+ES+BF		NR	Standardised reeducation program. Home: 5 days/week: no details. Weekly individual sessions	Strength & motor learning	1. ES vag 2. BF vag EMG. Clinic only	1. ES:15 mins 6s on/18s off, weeks 1–4, 8s on/24s off, weeks 5–8. 50 Hz, 250 msec. 2. BF 25 min	8 weeks	8

Dumoulin 2004 PFMT+ES+BF+ abdominal exercises		NR	a/a Additional weekly 30 min session with deep abdominal muscle training	a/a	a/a	a/a	8 weeks	8 + 8 group sessions for abdominal muscle training

Dumoulin 2004 Control group	weekly massage with PT						8 weeks	

Dumoulin 1995 PFMT+BF+ES	NA	1	5s/10 s, 10 reps, 2 sets Home: 4 sets/day Individual session with ES/BF 3x/week.	Maximal	1. ES Interferential current 4 suction electrodes 2. BF vag pressure Clinic only	1. ES 15 mins 10–50 Hz, 15 mins 50 Hz. 2. BF 15 mins	3 weeks	9

Finkenhagen 1998 PFMT	NA	1	6–8 s/6 s, 8–12 reps, 3–4 fast contractions at end of 'hold'. Home: 8–12 reps, 3 sets/day. Weekly exercise class (protocol as for Bo 1999)	Strength			6 months	1 individual + 24 group training

Glavind 1996 PFMT	NA	1	'standard procedure' – no details given. Individual sessions 2–3 times	NR			4 weeks	2–3

Glavind 1996 PFMT+BF	NA	NR	5–10s contractions, 10 reps in supine, sitting, standing, Individual instruction	NR	BF vag EMG + rectal pressure BF to avoid IAP rise	4 weekly sessions. Clinic only.	4 weeks	6–7

Hay-Smith 2004 PFMT ^a^	NA	NR	PFMT :motor relearning alone Home: no details	Motor learning			20 weeks	4 + 3 phone calls

Hay-Smith 2004 PFMT ^b^	NA	NR	PFMT: strengthening plus motor relearning. Home: no details	Strength & motor learning			20 weeks	4 + 3 phone calls

Johnson 2001 PFMT (SVC) +BF	NA	2. Vaginal perineo meter	10 s/10s, 15 minutes, submaximal (60% of MVC). 3 sets/day	Submaximal VoluntayContractions	BF vag pressure. Rectus EMG BF for first instruct ion	BF home training	6 weeks	2

Johnson 2001 PFMT (NMVC) +BF	NA	2. Vaginal perineo meter	10s/10s, 10 minutes, near-maximal (90% of MVC). 3 sets/day	Near-maximal Voluntary Contract ions	BF vaginal pressure Home trainer		6 weeks	2

Knight 1998 PFMT+BF	NA	1	Up to 10s/4s (individualised), fast (up to 10), up to 10 reps, 6 sets a day. 6–18 months:1 set/day	Maximal	BF vaginal pressure. Home trainer + clinic.	Home trainer: 1 set PFX per day. Clinic: weekly for 1 month, then bi-weekly. 6–18 months: BF 1/week	6 months	14

Knight 1998 PFMT+BF+ES (home)	NA	1	a/a	Maximal	As for PFMT+BF plus ES (home)	Vaginal, overnight, 10 Hz, 200 ms. 5 on/5 off. Low intensity. 6–18 months: BF 1/week	6 months	14

Knight 1998 PFMT+BF+ES (clinic)	NA	1	a/a	Maximal	As for PFMT+BF plus ES (clinic)	Vaginal, 16 × 30 min., 35 Hz, 250 ms. 5 on/5 off. High intensity, contraction with stimulator. 6–18 months: BF 1/week	6 months	14 PFMT+BF 16 ES

Miller 1998 PFMT	NA	1	Taught to contract and cough. Home practice.	Motor learning			1 week	2

Morkved 2002 PFMT	NA	1	6–8s/6 s, 8–12 reps (high intensity). 3–4 fast contractions at end of 'hold. Home: 3 sets/day. Individual sessions	High intensity			6 months	16

Morkved 2002 PFMT+BF	NA	1	a/a with home BF. Individual sessions	High intensity	BF vaginal pressure home trainer	6–8s/6 s, 8–12 reps (high intensity). 3–4 fast contractions at end of 'hold', 3 sets daily	6 months	16

Pages 2001 PFMT	NA	1	Group 5/week. Home: 100 reps/day during daily activities. Supine 10 mins, 2 sets/day. Group: different positions	'Isolated' contractions, intensity NR.			4 weeks group then 2 months home PFMT	3 individual + 20 group

Pages 2001 PFMT+BF	NA	1	1 group session, individual BF training 15 mins, 5/week/4 weeks. Home: 10 reps, 4 sets, 5 times/week	NR	BF vaginal pressure. Clinic only	15 min sessions Supine 10 reps/4 sets	4 weeks individual then 2 months home PFMT	23 individual

Parkkinen 2004 PFMT+ES+BF+VW	NA	1	Short, low-intensity, 8–10 reps. High intensity: 5s/10s, 5 reps, low intensity: 20–30s/40–60s, 5 reps, supine & standing. Contract & cough. Home: 2 sets/day, 5 days/week	High intensity	1. BF vag EMG 2. ES interferential 5–10 mins 50 Hz, 5–10 mins 10–50 Hz Clinic only. 3. VW	1. BF 2. ES 10 mins 3. VW (20–80 g), 30 min/day, 5 days/week, during daily activities	Weekly to one year. Duration individual-ised: 'until desired outcome achieved'	9 (3–29)

Parkkinen 2004 PFMT+VW	NA	1	a/a	a/a	VW	VW (20–80 g), 30 min/day, 5 days/week, during daily activities	12 months.	3

Pieber 1995 PFMT+BF	NA	1,2 Perineal ultrasound	Contract-relax times NR. 100 PFX per day. Encouraged to do the 'knack'. Individualised home program	Intensity NR. Relaxation of abdominals, gluteals, thighs	BF: Perineal ultra-sound (3 times). Clinic only	BF: Visualised PF on screen (3 sessions)	3 months	2–4 weekly intervals, 3 (asses ment)

Pieber 1995 PFMT+BF+VW	NA	1	a/a	a/a	1. BF: Perineal ultra-sound. Clinic only 2. VW	1. Visualised PF on screen (3 sessions) 2. VW (20–70 g) 15 mins during daily activities	3 months	a/a

Sung 2000 PFMT	Explanation, no treatment	NR	PFM exercises 'as developed by Bo', details NR. Exercises in clinic with video. Home: same exercises, details NR	Intensive			6 weeks	6

Turkan 2005 PFMT+ES	NA	2 (not clearly reported)	5s contractions, 10 reps, 5 sets/day, 5 sets added in each week. Home: also with activities of daily living, provocation	Maximum intensity	ES -Interferential, 4 vacuum electrodes Clinic only	10 mins each 0–10 Hz, 0–100 Hz. Voluntary contractions with ES	5 weeks	15

Wong 2001 PFMT+BF	NA	NR	Home: NR	Fast: maximal Slow: as long as possible.	BF vag EMG	5 sets: 'fast'/10s rest: 3 reps, 'slow'/1 min rest: 2 reps. with BF. Clinic only	4 weeks	4

Wong 2001 PFMT+BF+ abdominal EMG BF	NA	NR	a/a	a/a	BF vag EMG & EMG BF-rectus abdominis Clinic only	a/a with abdominal EMG BF to minimise rectus activity	4 weeks	4

PFMT = pelvic floor muscle training, PFX = pelvic floor exercises, BF = biofeedback, ES = electrical stimulation, VW = vaginal weights EMG = electromyography, vag = vaginal, reps= repetitions, NA = not applicable, NR = not reported, a/a = as above

#### • Pelvic floor muscle training

Studies were described by the broad types of PFMT which were employed, ie specific strength training (inducing muscle hypertrophy) or skill training (improving motor learning), and their exercise dosage (frequency, intensity, duration of the training programs and compliance) [10]. The effect of specifically activating or de-activating the abdominal wall during PFMT was investigated. While reducing abdominal muscle activity has been advocated to isolate the PFM and minimise intra-abdominal pressure (Laycock, 1994), more recently a synergistic activity of the deep abdominal muscles (transversus abdominis and lower fibres of obliquus internus) and PFM has been described [57-59]. Training of the deep abdominal muscles as a treatment for incontinence has been advocated [60] but more recently disputed [10].

#### • Biofeedback

Many different applications of biofeedback were described. Vaginal applications of EMG [32,36,38,41,42,50,51], pressure devices [44,45,47,48,56] or perineal ultrasound [49,53] were described. Three studies applied surface EMG BF on the surface of the abdominal wall as well to indicate abdominal muscle activity [32,42,50]. The EMG electrodes were placed over the rectus abdominis in one study [50] but the placement was not specified in the other two studies. Vaginal BF was used as a home treatment in three studies [44,45,47], as home and clinic treatment in one study [45] and in the others it was used only at clinic visits. One study [42] used additional rectal pressure BF to monitor intra-abdominal pressure.

Two studies used trans-perineal ultrasound to teach a correct elevating contraction at the first clinic visit [49,53] and in one study ultrasound was repeated for PFMT on two further occasions [49]. Another study [36] did not provide clear details whether pressure BF was used for teaching only or training as well.

#### • Electrical stimulation

Electrical stimulation was used in seven studies in different combinations of therapy. [33,39,45,51,52,54,56]. Three studies investigated PF/ES, four studies a combination of PFMT/BF/ES, and one study a combination of PFMT/ES/BF/VW. The application and protocols varied considerably. Two studies used interferential currents with externally applied suction cups with clinic treatment [52,56]. The others used vaginal application either with home stimulation [39,45] or at clinic visits [45,54].

#### • Vaginal weights

Different types of vaginal weights were used varying from 20 g to 100 g. Protocols required women to perform activities of daily living while retaining the weight in the vagina [37,49,51], while one [37] required women to perform 'gymnastics' in addition to routine daily activities but no details of this activity or of subjects' compliance were provided. In all three studies women additionally performed a PFMT program.

### Types of outcomes

A summary of the outcome measures used in terms of the ICS recommendations is presented in Table 12. Outcomes were reported under all categories except socioeconomic variables which were not reported in any study. However, in each category, different instruments were used or modifications of the same instrument. For example, in category 2 (quantification of symptoms by objective measures) the results of 19 pad tests were reported. Two were performed for 48 hours, two for 24 hours, one for 10 hours. In addition, eight different provocative pad tests with standardised bladder filling were performed [34,40,41,45,47,51,53,56] and another four 'standardised' pad tests were reported without details of either bladder filling or provocation [36,42,52,54]. One test using paper towel instead of a pad to quantify urine loss under coughing provocation was reported [46]. This variability precludes precise comparison of outcomes.

**Table 12:** Summary of outcome measures used according to ICS recommendations, need for surgery, side effects

Studies	C1	C2	C3	C4	C5	% subjects proceeding to surgery	No serious adverse events reported
Aksac (2003)		1	1	1			
Arvonen (2001)	1	1	1				
Balmforth (2004)	1	1		2			
Berghmans (1996)	1	2	1	1			
Bidmead (2002)	1	1		1			
Bo (1999)	3	3	1	1			yes
Bo (2000)				1			
Cammu & van Nylen (1998)	2	3	1	1		17	yes
Chen (1999)		2	1				yes
Dumoulin (1995)	1	1	1				
Dumoulin (2004)	1	1	1	2			yes
Finkenhagen (1998)	1						
Glavind (1996)	1	1					yes
Hay-Smith (2002)	1	2					
Johnson (2001)	1	2	1				
Knight (1998)	1	1	1				
Miller (1998)		1					
Morkved (2002)	2	2	1	1		4.3–6.3	yes
Pages (2001)	1		1				
Parkkinen (2004)	1	1	1			10.5	yes
Pieber (1995)	1					0	
Sung (2000)	1						
Turkan (2005)	1	3	1				
Wong (2001)	1	2	1	1			

ICS Outcome Measurement categories; C 1= patient symptoms: perception of cure/improvement; C 2 = quantification of symptoms (objective measures): pad use, diary of incontinent episodes, pad tests; C 3 = clinicians' measures (pelvic floor muscle measures); C 4 = quality of life measures; C 5 = socioeconomic measures, blank cells indicate no relevant report.

**Figure 1:**
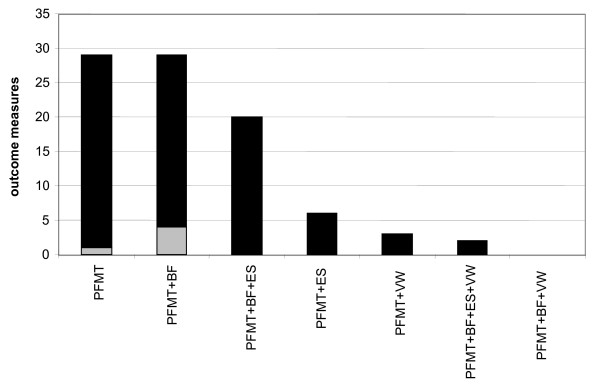
**Summary of incontinence outcomes for different combinations of physical therapy**. Total number of positive and statistically significant measures of incontinence (black) and non-significant measures of incontinence (grey) for different combinations of physical therapy. Included are subjective, objective and quality of life measures. PFMT = pelvic floor muscle training. PFMT+BF = pelvic floor muscle training with biofeedback. PFMT+BF+ES = pelvic floor muscle training with biofeedback and electrical stimulation. PFMT+ES = pelvic floor muscle training and electrical stimulation. PFMT+VW = pelvic floor muscle training with vaginal weights. PFMT +BF+ES+VW = pelvic floor muscle training with biofeedback, electrical stimulation and vaginal weights. PFMT+BF+VW = pelvic floor muscle training with biofeedback and vaginal weights.

A summary of all the positive and statistically significant (p < 0.05) and the non-significant measures of effect for each category of study (PFMT, PFMT/BF etc) is presented in Figure 1. Each measure is displayed for within-group or, if there was a no-treatment control group, also for between group differences.

### Psychometric properties

None of the level III & IV studies and nine of the 16 level II studies included statements about the reliability and validity of the outcome measures used [see additional files 4 &5]. The use of outcome measures which are valid, reliable and sensitive to change is vital when considering the effects of treatment in order to detect valid changes which are greater than measurement error [61]. Caution must be exercised when considering the results of studies where valid and reliable outcome measures have not been used.

### Outcomes in terms of cure/improvement

The definitions used for 'cure' and 'improvement' varied widely and are listed in Table 13. Five studies [33,39,50,51,53] did not report their outcomes in terms of the numbers (percentages) of subjects who were cured/improved at all. All estimates of 'cured' and 'improved' are expressed as the percentage of subjects who completed treatment compared with the number who started treatment. The number (percent) of withdrawals is presented to permit estimates of bias.

**Table 13:** Definitions of 'cure' and 'improvement'

Definitions of cure	Studies	Definitions of Improvement	Studies
Less than 1 g loss on pad test	Parkkinen (2004), Dumoulin (1995)	Decrease of 50% or more in pad weight	Aksac (2003)
1 g or less on pad test	Aksac (2003) Glavind (1996)	Self-report of less urine loss compared with pre-treatment	Pieber (1995)
Less that 2 g loss on pad test (st.b.vl)	Dumoulin (2004) Knight (1998)	Self-report: continent (cured), almost continent (improved) (5 point Likert scale)	Bo (1999)
2 g or less on stress test (st.b.vl)	Bo (1999) Morkved (2002)	Rare or minor incontinence on exertion & 'satisfied'	Chen (1999)
Self-report: unproblematic (5 point Likert scale)	Bo (1999) Morkved (2002)	Decrease of > 50% in IE & decrease in 'symptoms'	Pages (2001)
'No incontinence' (measure NR) & no incontinence on UDS	Chen (1999)	Greatly improved: >75% improvement on pad test	Knight (1998)
No incontinence for 7 days	Johnson (2001)		
No urine loss on any occasion & negative stress test	Pieber (1995)		
No urine loss on paper towel test	Miller (1998)		

IE = incontinent episodes, st.b.vl. = standardised bladder volume, UDS = urodynamic studies

### Other outcomes

Four studies reported on the numbers of women who had surgery either during the study or after completion of treatment [32,47,49,51]. Ten studies reported on the occurrence of any adverse events as a result of treatment [34,41,42,46-49,51,54,55].

#### 1. What is the evidence for PFMT, either alone or in combination with adjunctive therapies, when considering all treatment protocols, for the treatment for SUI in women, in the short and medium terms (up to 12 months after treatment)?

##### 1.1 PFMT alone

Twelve 12 RCTs with 13 treatment arms, one level III-2 and one level IV studies investigating PFMT protocols were identified (Table 4). Cure rates ranged from 2% [43] to 75% [36,43] and rates of cure/improved ranged from 41% [43] to 100% [48]. However, when considering the evidence from the two studies with >90% quality scores [34,47], reported cure rates were 44% to 57% and 'cure/improvement' rates from 48% to 93%, depending on the definition of cure/improvement. These two studies demonstrated treatment effects based on 13 different measures of outcome. Both reported pad test and self-report of symptoms giving conflicting findings. Bo (1999) reported a higher cure rate with subjective assessment (56%) while Morkved (2002) reported a higher cure rate with objective assessments (46% with a short provocative pad test and 57% with 48 hour pad test). Direct comparisons between study outcomes are to be considered with caution due to the range of definitions of cure and improvement reported.

No adverse events were reported as a result of PFMT [34,42,46,47,55]. Two studies reported the number of subjects having surgical intervention either during (4.3%) [47] or at the end of the study (17%) [32].

Considering all study designs, 28/29 (97%) different measures of incontinence reported a positive and statistically significant change. Thus in considering the strength of evidence for PFMT, there is strong evidence from a number of high quality level II studies, with consistently positive and significant findings, based on multiple measures of outcome that PFMT is effective for women with SUI.

##### 1.2 PFMT with BF

Ten RCTs with 12 study arms (quality scores: 96% [47] to 39% [36]) and one level IV study were identified reporting the outcomes of PFMT combined with BF training (Table 5). Rates of cure from 22% [49] to 80% [36] and rates of cure/improvement from 86% [49] to 100% [36,48] were reported. The highest quality study using BF demonstrated a cure rate of 58% (provocative pad test) and of 62% (48 hour pad test) for women training at home with pressure BF [47]. A combined rate of 97% cured/improved was reported (self-report). There was no statistical difference in the outcomes of women in the other arm of this study performing an identical intensive PFMT program over 6 months without BF. Four studies using vaginal EMG BF as a clinic treatment showed cure rates from 25–80% [32,36,38,42] or positive and statistically significant outcomes [50].

Regarding the use of EMG BF on the abdominal wall, one study found no difference in outcome with the addition of abdominal wall BF to reduce rectus abdominis activity [50]. Another also used surface EMG to reduce abdominal muscle activity [44], but the heterogeneity among the protocols and lack of information about electrode placement precluded conclusions about its value. There was also insufficient evidence from this review about the role of ultrasound to teach or train a PFM contraction in order to make any recommendations.

One study reported that no subjects underwent surgery during the study period [49]. Another reported that 3/48 (6%) of women proceeded to surgery after unsuccessful treatment [47]. There were no reports of the occurrence of adverse events [42,47-49].

When considering all the studies on PFMT/BF, a total of 25/29 (86%) incontinence outcomes were positive and statistically significant, while four outcomes failed to show significant change after treatment. All of these occurred in two studies [44,50] with treatment times of 4 and 6 weeks respectively. Non-significant results may have been due to measurement error, as pad tests without demonstrated reliability were used [44,50] and because of the short duration of training, which may have been insufficient to effect physiological changes. Type II error should also be considered when interpreting these results as one study [50] gave no evidence of a power calculation to ensure sufficient numbers to demonstrate a treatment effect. Thus, in summary, there is strong evidence from a number of RCTs that PFMT with vaginal EMG or pressure BF is effective for the treatment of SUI, but it may be no more effective than PFMT alone.

##### 1.3 PFMT with ES

There was evidence from one level II study (quality score 43%) [39] for a treatment effect using a combination of PFMT/ES, although no cure rates were reported. No difference between groups was found when home treatment with vaginal ES was added to a 14 week PFMT program, but there were positive and significant within-group differences for PFMT/ES based on objective and quality of life measures. This study was only available as an abstract, thus the potential exclusion of useful information may have contributed to the poor quality score. When including the non-RCTs, all measures of incontinence (6/6) showed positive and statistically significant change after treatment. One study [54] reported no adverse events. Thus there is limited evidence from one RCT that PFMT combined with vaginal ES is an effective intervention for women with SUI, but it may be no more effective than PFMT alone.

##### 1.4 PFMT with VW

One level II study (quality score: 65%)[37] and one level III-2 study [51](quality score: 74%) provided evidence about PFMT combined with vaginal weights (Table 7). Arvonen (2000) reported cure rates of 50% (pad test) and 22% (subjective report) and cure/improvement rate of 61%. This study compared women training the PFM with and without VW, but with a different training protocol for each group. Across both studies, all measures of incontinence (100%) showed positive and statistically significant change after treatment.

One study [37] reported no pain associated with using VW and a dropout rate of 12%. The other study [51] reported that four subjects proceeded to surgery for their incontinence during the study period.

There is evidence from one RCT that PFMT with vaginal weights may be effective in improving the outcomes for women with SUI. However, from this review, it is not possible to comment whether PFMT with VW is more effective than the same PFMT protocol performed without VW.

##### 1.5 PFMT with BF/ES

One level II study (quality score: 91%) [41] with two arms using the same combination of PFMT with vaginal EMG BF/ES, one arm with the addition of an abdominal muscle training program, showed cure rates of 70% & 73% respectively and a cure/improvement rate of 90% in both arms. A further level II study (quality score: 83%) [45], using two different types of ES ('low' intensity at 10 Hz and 'high' intensity at 35 Hz) in combination with PFMT/BF, reported combined cure/improvement of 67% when based on intention to treat. A level IV study (quality score 68%) [56] used a combination of PFMT with vaginal pressure BF and interferential currents for ES (Table 8). Overall, 20 different incontinence measures were reported, all exhibiting positive and statistically significant change.

When assessing the effect of adding ES to PFMT/BF, one study found no statistically significant difference in pad test results or PFM strength between groups, suggesting no additional benefit [45]. However, as no power calculation was reported, these results should be interpreted with caution because of the possibility of insufficient subject numbers.

There were no reports of adverse events and no statements were made regarding surgical intervention. However, one study reported women withdrawing from home treatment with ES because of discomfort [45].

Thus there is good evidence from two level II studies that PFMT combined with BF and ES is effective treatment for women with persistent postnatal SUI and also for older women up to the age of 68 years. Due to the heterogeneity in the protocols, it is not possible to identify which components of the programs contributed to their efficacy.

##### 1.6 PFMT with BF/VW

One level II study (quality score 57%)[49], using this combination of therapies, was identified for this review (Table 9). Trans-perineal ultrasound was used to provide BF to identify and reinforce a correct elevating contraction of the PFM at three clinic visits, with PFMT including VW for home training. The reported cure rate was 39%, the combined cure/improvement rate was 85%, but no clinical outcomes were reported in terms of statistical significance. There is thus limited evidence from one level II study for this combination of treatments.

##### 1.7 PFMT with BF/ES/VW

No level II studies were identified but one level III-2 study (quality score 74%)[51] included in this review had a treatment protocol with PFMT, BF, ES and VW (Table 10). Cure rates at the end of the 12 month study period were not reported but both measures of outcome showed positive and statistically significant change after treatment. Outcomes were reported at 5 years but there was co-intervention and contamination of the treatment groups after 12 months which precluded group analysis. Thus there is only limited evidence from one non-RCT for this combination of treatment.

Three studies involving ES which considered adverse events reported none with combined PFMT/ES [41,51,54].

##### 1.8 Length of follow up

Follow-up after the end of the treatment program was reported by two RCTs [42,45] and two non-RCTs [51,54] in this review. One RCT suggested that urine loss on pad testing was reduced between end of intensive treatment and 6 month follow-up all in groups but statistically significant differences were not reported [45]. The other RCT assessed women after 4 weeks of treatment, again two months later and after 30 months by postal questionnaire. Women who had trained with BF were reported to have better continence status than women performing PFMT without BF [42]. Of the two non-RCTs, one evaluated women four more times over 21 months after three months of a PFMT/ES program [54]. Declining success over this time was reported, corresponding with decline in PFM exercise compliance. The other study suggested ongoing benefit 5 years after a combined PFMT/VW program [51]. However, the results of studies of lower methodological quality should be interpreted with caution.

#### 2. What is the evidence for different types of PFMT?

##### Strength training

The recommended exercise dosage for strength training of the PFM has been extrapolated from exercise physiology principles for normal skeletal muscle. Slow velocity, near maximal contractions, sustained for 6–8 seconds, with 3 sets of 8–12 contractions performed 2–4 days a week and continuing for up to 5 months, are recommended [10,16].

##### • Effect of strength training on incontinence outcomes

Three level II studies [34,44,47], one level III-3 [33] and one level IV study [55] investigated a training protocol with maximum sustained PFM contractions as the only type of PFMT. Some women trained with BF [44,47]. The duration of the training period varied from 6 weeks [33,44] to 6 months [34,40,47,55]. All but one [44] were otherwise based on a similar exercise dosage in terms of the intensity, number of repetitions and frequency of training, as recommended by Bo (2004)[10]. All the studies required the women to train daily at home. However, there were differences in the protocols: two studies had an additional weekly group session over 6 months [34,55], where another had weekly or fortnightly therapist contact over 6 months but without group training [47].

The reported efficacy of these strength training protocols from the two high quality studies (quality score >90%) was 44% & 56% [34] and 58% & 40% [47] in terms of the number of subjects cured by objective and subjective measures respectively at 6 months. Rates of cure/improvement were higher: 48% [34] and 93% [47] but were based on different self-rated assessment scales, which may partly explain the discrepancy in outcome. One RCT [44] reported 38% of subjects subjectively cured at 6 weeks.

There is evidence from two high quality level II studies that PFMT according to strength training principles is effective in relieving the symptoms of SUI in women. Change in symptoms may be noted after six weeks. Effective outcomes were achieved with either additional regular group training or individual sessions with the physiotherapist.

##### • Effect of strength training on PFM strength

Possibly the most valid and reliable measure of PFM strength was reported by Dumoulin (2004) using a dynamometer. Although changes in incontinence were demonstrated after 8 weeks of PFMT with clinic-based BF/ES, there were no statistically significant increases in PFM strength in either arm of this study. Other studies reported PFM strength changes using perineometry [33,34,36,44,45,47,48,50], which may be a reliable but not necessarily valid measure due to influences of intra-abdominal pressure [62]. One RCT showed an increase in PFM strength after 4 weeks of PFMT [50] and another after 3 months [47]. Three RCTs demonstrated increased strength after 6 months of an intensive strength training protocol [34,45,47]. One showed incremental increase between 0–3 and 3–6 months [45]. Some training was done with BF [44,45,47,50]. One RCT demonstrated strength changes after 6 weeks of submaximal PFMT [44], an intensity which has been shown to increase muscle strength in untrained individuals [10]. However, no data was provided about prior PFMT in the subjects to substantiate this in the study population.

One study used perineal ultrasound to demonstrate a statistically significant elevation of the bladder neck position after PFMT for three different conditions: at rest, with maximum Valsalva, and maximum contraction [53]. Two RCTs [36,37] reported PFM strength changes using digital assessment but this measure has doubtful reliability for scientific purposes [62].

In summary, there is strong evidence from a number of high quality RCTs that using a specific strength training protocol increases PFM strength, with measurable changes between 4 weeks and 6 months. However, in accordance with physiological principles [10], evidence from this review confirmed that longer training times produce greater gains in strength.

##### Skill training

In terms of PFMT, skill training implies the acquisition of a higher level motor skill in timing a PFM contraction just prior to the event which provokes urine loss. This approach to PFMT has been variously called motor learning, motor re-learning, the 'knack', functional training and counter-bracing [10].

Two RCTs investigated the effect of teaching women with SUI to contract the PFM just prior to a rise in intra-abdominal pressure [43,46]. One tested women after one week of practising the 'Knack' of contracting the PFM before a cough, with reported cure rates of 23% (with a deep cough) and 75% (with a moderate cough) [46]. The other study reported 7% of subjects cured and 47% cured/improved, using a more complex functional training protocol, although details were not reported [43]. This study reported no difference between two groups training with a skill training protocol and with combined strength and skill training. However, the authors attributed the non-significant result to type II error.

Nine other studies included some aspects of skill training as part of their PFMT protocol, but details of the actual training process and the exercise dosage were poorly reported [32,37,38,41,48,49,51-53].

While there is increasing evidence that skill training may be an important component of a PFMT protocol, there was insufficient information provided about the specific exercises performed to recommend any particular approach to skill training.

##### Combination strength & skill training

Six studies were identified which included both maximum intensity contractions and elements of skill training in their PFMT protocols [37,41,43,51-53]. Three of these were RCTs with very different treatment protocols and outcomes [37,41,43]. Dumoulin (2004), with the shortest duration of 8 weeks training and weekly contact for training with the physical therapist, had the highest reported cure rate (73%). Arvonen (2000) reported 50% cure using strength training as well as vaginal weights for additional skill training during physical activities. Evidence from these studies suggests that a combination of strength and skill training is effective treatment for SUI but the contribution of each component to the outcome is unclear.

##### Role of abdominal muscles

Dumoulin (2004) investigated the effect of adding specific deep abdominal muscle training to a combined PFMT/BF/ES program and found that it conferred no statistically significant benefit. By contrast, Wong (2001) investigated the effect of reducing activity of the rectus abdominis during PFMT using surface abdominal EMG BF but found no benefit with objective measures.

Four other studies in this review [32,36,44,49], specifically trained relaxation of the deep abdominal muscles, while one other stated that training of the deep abdominal muscles was included in weekly group sessions [34]. However, the different methods of assessing outcome and multiple other confounding variables do not allow conclusions to be drawn from these results.

In summary, thus there is evidence from one high quality RCT study to suggest that the addition of deep abdominal muscle training confers no additional benefit for women performing a combined PFMT/BF/ES program.

#### 3. What other reported factors could affect outcome of physical therapy?

##### Age

Women from age 18 to 84 were included in the 24 studies in this review, suggesting that women of all ages can be expected to respond to physical therapy. There was evidence from high quality RCTs for specific training programs for young women [41] and mid-aged women [34,47]. One study showed that skill training was effective in older women [46] but evidence is lacking for other specific physical therapy programs specifically for older women.

##### Initial severity of incontinence

Not all studies reported initial severity of incontinence symptoms but in those which did, two different measures were used: number of incontinence episodes per day [34,38,44,54] or week [32,50] and the volume of urine lost on pad test [34,36-47,50,52-54,56]. Due to the differences in pad test methodology it was not possible to make direct comparisons between populations at baseline.

A number of the RCTs stratified women to the treatment groups to remove the confounding effect of severity of baseline symptoms of incontinence, although none reported subgroup results. However, one study found that women with more mild symptoms of SUI responded better (88% cure) to the same treatment program than women with severe symptoms, none of whom were cured [52]. Although women in that study were not randomised but assigned to groups according to severity of symptoms, baseline variables of age and BMI, which could have been confounders, were not statistically significantly different between groups.

##### Compliance with the training program

The effectiveness of an exercise program can only be evaluated if it is known how well the subjects complied with the prescribed home program. Seven studies in this review reported on subject compliance with the treatment protocol [34,39-41,45,54,56]. In all cases but two [45,54] it was reported that a diary was kept. One study found that compliance with the home PFMT protocol predicted a successful outcome [54]. Three studies [34,39,45] reported the actual level of subjects' compliance. In groups with only PFMT as a home program, it was reported that 75% [39] to 93% [34] of subjects were compliant. One study reported that subjects performing a home PFMT program with daily pressure BF over 6 months were compliant with the program 75% of the time, while only 48% were compliant when home ES was added to the home treatment program [43]. Another study reported good or excellent compliance by 45% of subjects when combining ES with PFMT in a home program [39].

In summary, compliance with the training program was not routinely reported. Despite the lack of a standardised approach to assess and report compliance, it appears that compliance may be greatest if a home program does not include BF or ES.

##### Initial pelvic floor muscle strength

Although all studies reported teaching women to contract the PFM correctly prior to commencing a PFMT program only one stated that all women were actually able to do so [48]. One study included women who were initially unable to contract their PFM but did not report numbers of affected women or the effect of this on the outcome [42]. Turkan (2005) assigned subjects to three groups according to severity of incontinence by pad test results and reported significantly lower PFM strength in the women with most severe incontinence (>10 g on pad test) before treatment. Even though no women were cured after treatment in the most severely affected group, this group had the greatest response to treatment in terms of changes in PFM strength and leakage on pad test. Similarly, Knight (1998) reported that initially lower PFM strength on perineometry was correlated with greater improvement in continence outcomes.

#### 4. What is the evidence for the optimal period of treatment and number of treatments?

##### Duration of treatment period

Parkkinen (2004) reported a mean of 9 (3–29) weekly treatments with subjects ceasing treatment when a 'desired outcome' was achieved. All the other studies had a treatment protocol with a predetermined training period and number of contacts with the therapist. The length of treatment varied from one week [46] to 24 months [54].

##### Number of treatments

The number of treatments varied from two [46] to 30 [34,40]. The number of treatments was not stated in two studies [39,53] but was standardised in all other studies except Parkkinen et al (2004). Instruction was provided in groups as well as individually (see Table 11 for details).

##### Response time

One study [46] showed a change in incontinence status after only one week using a skill training approach, while another [56] reported changes after 3 weeks. Nine studies, all reporting positive and statistically significant change in symptoms, had training periods from 4–8 weeks 9 [33,36,38,41,42,44,48,50,52], while others ranged from 3–6 months [32,34,37,39,40,43,45,47-49,53-55]. From this review it is not possible to determine if there is an optimal length of treatment period or number of treatments. However, one level III study showed that women respond at different rates to the same treatment protocol [52].

#### 5. What is the evidence for the effectiveness of physical therapy in the clinical setting?

Only one study stated specifically that the intervention was performed in a physiotherapy clinic in a primary health care setting [55]. This level IV study found that 67% of subjects with SUI were cured/improved after six months of PFMT with a trained physiotherapist, suggesting that outcomes in clinical practice may comparable with those of RCTs.

##### Generalisability of findings to clinical practice settings

There was little information provided in the studies reviewed about factors relevant to determination of the generalisability of the study findings, for example, the setting where the treatment took place, the source population for patients or how the patients were selected. In eight studies [37,38,45,48-52], treatment was conducted in a hospital or university outpatient clinic but in 14 studies location was not stated. One was a multi-centre study but the settings were not identified [34]. The profession of the person performing the treatment was stated in 19 studies (all physiotherapists) but it was not clearly stated in the other five studies [33,36,42,44,46].

## Discussion

This systematic review reports the evidence of physical therapy interventions for SUI from full text studies or abstracts published in English during the last decade. Despite suggestions that the methodological quality of studies has increased over time, no correlation was found between a more recent date of publication and the quality score of the studies published over the last 10 years and included in this review. Thus it must be acknowledged that high quality studies published prior to 1995 may have been missed by the limitations on publication date which were set.

The inclusion of both RCTs and non-RCTs dictated the presentation of results as a narrative summary. The methodological quality of the studies was variable, with some RCTs being of lower quality than the lower level studies. This provides a dilemma for systematic reviewers, as restriction of study inclusion to RCTs is considered to ensure identification of high quality studies [20,63]. However, the possibility of well-designed cohort studies providing less biased evidence than poorly designed RCTs has been documented [64]. It is acknowledged that the methodological quality of the critical review tools themselves may have incorrectly reflected the quality and ranking of the included studies [65].

One of the aims of this review was to investigate outcomes relevant to clinical practice. To this end, level III and IV studies, not previously reported in systematic reviews of the literature on SUI, were included. The inclusion of these studies with lower levels of evidence provided information about aspects of physical therapy not obtainable from the RCTs reviewed, for example, about the different response rate and the effectiveness of treatment in the primary care setting.

### Question 1: What is the evidence for PFMT, either alone or in combination with adjunctive therapies, when considering all treatment protocols, for the treatment for SUI in women, immediately and up to 12 months after treatment?

This review found consistent evidence from high quality level II studies for PFMT alone and in combination with adjunctive therapies in the treatment of SUI. Further evidence is presented about the efficacy of PFM strength training, in support of previous reports [14,16]. New evidence is provided for the efficacy of different combinations of PFMT with BF and ES but the combination of PFMT with BF was shown to be no more effective than PFMT alone. It is unclear specifically how the combinations of therapy contribute to the outcome of any training program and whether it is more effective to administer adjunctive therapies in the clinic setting or home environment.

All of the studies reviewed demonstrated positive treatment effects for physical therapy, despite a range of training protocols and combinations of adjunctive therapies. Studies with a lower quality score have a greater potential for bias and, with the plethora of different outcome measures used, it was not possible to directly compare the effectiveness of the different protocols. Four papers were only available as abstracts so that the assessment of methodological quality in these studies may be underestimated due to the limited information available.

#### Factors not assessed by the studies which could affect outcome

This review found that physical therapy is effective in the treatment of SUI. However, there were other factors, common to all studies, which may have contributed to the differences in outcome. The expertise of health professionals may vary and also the quantity and quality of the educational information about the condition and PFM function. The impact of these factors on the outcome of treatment has yet to be evaluated. Furthermore, it has been well documented that many women depress the PFM instead of contracting it in a cephalad direction after brief verbal or written instruction [66,67]. Thus assessment for correct action by vaginal examination should be considered a prerequisite for commencing a PFMT program. However, correct action was not always reported and several studies used other methods (vaginal EMG or pressure BF) which are not considered to be valid assessment tools [62]. Two studies used perineal ultrasound, which has demonstrated reliability but is not a readily available clinical tool [62]. However, the reliability of any method will be dependent on the experience and expertise of the user and the results should be interpreted with this in mind [68].

#### Outcome measures

The plethora of outcome measures reported in the included studies also contributed to the difference in results and constrained comparisons between studies. Outcomes measures have been reported here in terms of their positive and statistically significant findings and also reported in terms of the recommended ICS categories. It was notable that outcomes were reported under every ICS category except socio-economic outcomes. Previous systematic reviews [14,16] have noted the absence of reports on socio-economic outcomes. This review substantiates this finding for the past decade.

Not all studies reported their outcomes in terms of the number of subjects 'cured' or 'improved', although this would seem to be an important consideration in determination of the clinical effectiveness of any intervention for this condition. Moreover the definition of 'cure' has not been agreed. Different methods of evaluating 'cure' eg by pad test and self-report resulted in different outcomes. This difference may be explained by the fact that women, who are provoked to leak during a stress test which involves vigorous jumping, but who do not normally engage in jumping, may report satisfaction with treatment outcome. This might suggest that patient self-report and satisfaction with treatment are possibly more relevant measures. However, very different cure rates are obtained if women are asked to report if they are continent (as opposed to 'almost continent') or if their incontinence is 'unproblematic'. This language difference possibly accounted for the considerable difference in cure/improvement for two otherwise similar PFM strength training programs. The use of common, standardised self-report questionnaires is recommended in research and clinical practice by the ICS, and if utilised, will facilitate interpretation and comparison of future studies.

Reported cure rates were much lower than the percentages of women 'cured & improved'. This was also noted by Hay-Smith et al (2001). If the small percentages of women seeking surgical treatment after physical therapy for SUI are considered as a measure of success, then it would seem that the greater measure of effect, 'cured & improved', may be a more valid expression of women's satisfaction with the outcome. However a validated, ICS-approved satisfaction score is currently lacking.

There was little evidence about outcomes in the medium term up to 12 months after the completion of treatment. It was not the aim of this review to consider the longer term outcomes of physical therapy. However, outcomes in the short, medium and longer term are important information, both for consumers and for the calculation of the economic benefits of physical therapy particularly when compared with alternative treatments.

### Question 2: What is the evidence for different types of PFMT?

There is strong evidence from a number of high quality RCTs for specific strength training of the PFM in effecting change in continence status, underpinning its theoretical rationale and confirming previous reports [14,16]. There is evidence that PFM strength continues to increase over six months with specific strength training. Changes in bladder neck position as a result of PFMT have been demonstrated, suggesting structural changes in the PFM. However, the optimal training protocol is less clear as different approaches were effective. Thus the addition of weekly group exercises or individual sessions with the therapist may not be essential components of the training per se but rather the training effect may be enhanced through regular therapist contact for motivation.

Despite the number of studies including skill training in the PFMT protocol, its contribution in effecting change in health outcomes was not clear. There was considerable heterogeneity among the treatment and training protocols, precluding determination of clear conclusions. However, from the review, it appears there is sufficient weight of evidence to recommend a combination of strength and skill training in the treatment of SUI.

It should be remembered that only studies of PFMT for women with SUI were included in this review. It was not the aim of this review to consider the evidence of all the available literature on the effect of PFMT on different parameters of PFM function such as strength, endurance or skill level for women with other types of PFM dysfunction or for asymptomatic women. Therefore the effects of the PFMT protocols described may not be shown in other populations of women, particularly in those with other dysfunctions of the PFM such as prolapse and bowel incontinence.

This review found very different approaches to training the abdominal wall muscles in conjunction with the pelvic floor. There were no trials where deep abdominal training alone was performed as an intervention for SUI. However, the outcomes of an effective PFMT program were not improved by the addition of deep abdominal muscle training, nor by reduction of rectus abdominis activity by surface EMG BF.

The evidence from this review, that there is no benefit in adding BF, ES or deep abdominal muscle training to a PFMT program, should be considered from a clinical perspective. There may have been subgroups of women with different characteristics who responded differently to the treatment protocol but who were not identified in the analysis. In clinical practice, patients have different characteristics which will demand a reasoned approach to the choice of treatment at any one time. Thus it cannot be assumed that additional deep abdominal muscle training may not be useful for selected women with SUI who have demonstrated weakness of their deep abdominal muscles or that BF may not be beneficial for some women with poor proprioception of their pelvic floor or low motivation to exercise. It seems vital for the clinician to consider all relevant clinical findings (eg age, baseline pelvic floor muscle strength, proprioception, motivation, general physical fitness) when deciding on the best treatment for any one patient.

### Question 3: What other reported factors could affect outcome of physical therapy?

#### Age

This review found evidence for PFMT with and without adjunctive therapies for women up to the age of 84 who suffer SUI. There was evidence from a number of RCTs for the efficacy of a specific training program with PFMT, BF and ES for younger women after childbirth. There were a number of RCTs with consistent reports of efficacy of PFM strength training in women of mid-age, but limited evidence for specific PFMT protocols for older women. Given the demographics in the western world with increasing numbers of women living longer and the known association of incontinence with increasing age, effective training programs for older women are needed.

#### Initial severity of incontinence

Previous studies have reported conflicting findings about the effect of initial incontinence severity on the outcome of treatment [14,16]. The results of this review suggest that although fewer women with more severe symptoms may be cured by physical therapy, there may nevertheless be a significant improvement in their symptoms. Whether women with more severe SUI require longer treatment, different PFMT protocols or different combinations of therapy remains to be determined.

#### Compliance with the home training program

Another factor which may influence outcome is the degree to which subjects actually comply with the treatment program prescribed. Compliance with PFMT is a complex issue and has been the subject of a previous review [69]. The terminology is not agreed as some authors consider 'adherence' to be a more appropriate term implying voluntary co-operation rather than coercion [69,70]. Subject compliance or adherence was infrequently and generally poorly reported with no standardised, validated or reliable approach to its assessment. However it would appear to be of considerable importance in any PFMT program which depends on subjects performing exercise in order to effect physiological changes. There are complex psycho-social issues involved in interventions which demand that women commit time and effort on a regular basis to training [69,70]. It is likely in the high quality studies with good outcomes that subjects adhered to the treatment protocol. However, in studies which reported poorer outcomes and also did not report subjects' compliance, it is not possible to say whether an ineffective intervention or the subjects' lack of compliance was responsible for the poor result.

#### Initial PFM strength

There was evidence from two studies suggesting that women with weaker PFMs had a greater improvement in continence symptoms than women with stronger PFM. Previous reviews have reported conflicting findings [14,15]. There were no reports of what strategies were used if women were unable to contract the PFM at all, even though this would be likely to have an adverse effect on outcome.

### Question 4: What is the evidence for the optimal period of treatment and number of treatments?

We found evidence for the efficacy of shorter treatment protocols than the 4–6 months recommended by the ICS. The basis of the ICS recommendation was to allow time for an increase in PFM hypertrophy and volume as essential processes for increasing muscle strength. However, this review has shown that treatment programmes of less than three months may result in improved continence status as well as increased PFM strength. Whether the combination of PFMT with adjunctive therapy or the actual exercise dosage is the critical factor is unclear. The optimal length of treatment and the number of treatment episodes could be useful information for the marketing of physical therapy for SUI. Some women may be deterred from starting a physical therapy program if told that it is necessary to commit to six months of intensive training with weekly classes in order to become dry. This could be the focus of future research as it seems important information for consumers not only because of the implications for their time commitment and motivation but also because of the cost. More precise information about the length of treatment and frequency of therapist contact would underpin economic evaluations of conservative treatment which are currently lacking.

### Question 5: What is the evidence for the effectiveness of physical therapy in clinical practice settings and can the findings in the research settings be generalised to clinical practice?

This review sought to determine the effectiveness of physical therapy in the clinical practice setting where treatment is administered to a regular clinical population by continence practitioners. Only one study clearly took place in a clinical practice setting but as the inclusion criteria were not stated in the abstract, it was not possible to identify the characteristics of the study population. However, it appears that PFMT conducted in a primary care setting may be effective for the treatment of SUI.

The other studies in the review were considered for the generalisability of their findings to clinical practice by identifying the patient populations from which the study samples were drawn, the types of settings in which treatment was carried out and the health professional performing the treatment. However, this information was generally poorly reported so that only limited conclusions can be drawn.

Physiotherapists were the only health professionals stated to be performing the treatment (in 83% studies), and while continence training can be assumed for the therapists in these studies, the level of expertise is likely to be a key factor in determining success. Expertise in continence management is likely to be a more important factor influencing outcome in studies of clinical practice and should be considered a pre-requisite for health professionals treating SUI.

The effect of selection bias should also be considered in this context. Bias is potentially introduced when a study population consists of volunteers, who may be particularly motivated and compliant. Volunteers may be well motivated to succeed, particularly in studies requiring commitment to a daily exercise program over a lengthy period of time. Thus the outcomes of studies with a sample of volunteers may overestimate the true treatment effect. All three of the highest quality studies had study populations consisting at least partly of volunteers. In clinical practice, women referred for treatment may be variable in their enthusiasm about committing to a lengthy exercise program. Thus there may be some limitations to the generalisability of the results of RCTs recruiting volunteers and this should be considered by clinicians when interpreting the results.

## Conclusion

### Implications for practice

• There was strong evidence that PFMT alone, with BF and with ES/BF is effective for women with SUI, with expected rates of cure up to 73% and cure/improvement up to 97%.

• There was strong evidence for strength training of the PFM to reduce symptoms of SUI and to improve PFM strength.

• Changes in incontinence outcomes were demonstrated after treatment duration of one week to six months, but improvements in PFM strength may require at least 3 months of specific strength training.

• No benefit was found in this review in adding BF, ES or abdominal muscle training to a PFMT protocol. However, it is likely that these interventions still have a place in clinical practice as adjuncts to PFMT in particular populations of women.

• Strength PFMT protocols were effective in younger and mid-aged women, but there was scant evidence on strength training in older women.

• Evidence for skill training was found, especially if combined with strength training in women of all ages, but the optimal specific training protocol for skill training is unclear.

• Women with different severity of symptoms and initial PFM strength require different training programs and protocols. Women with weaker initial PFM strength and more severe symptoms may have the greatest percentage improvement in symptoms.

• Subjects using BF or ES as home treatment may be less compliant with a treatment program than women performing PFMT alone.

• No serious adverse events have been reported with physical therapy.

### Implications for research

Research is needed into:

• economic outcomes as none have been reported

• the effectiveness of physical therapy in routine clinical practice settings

• the external validity of RCTs. Future studies should more adequately describe the setting for the intervention, expertise of person delivering the treatment, the source and characteristics of subjects

• the longer term outcomes of physical therapies

• programs and protocols appropriate for different subgroups of women eg women of different ages and with different severity of incontinence

• the factors which influence a subject's likelihood of attending appointments, continuing with treatment and complying with the home training program

• the optimal length of an episode of care

• a more standardised approach to outcome measurement in research with appropriate outcome measures reflecting clinical practice requirements

• an optimal minimum set of common outcome measures relevant to research and clinical practice settings

## Competing interests

The author(s) declare that there are no competing interests.

This study was supported by the Centre for Allied Health Evidence, University of South Australia.

## Authors' contributions

PBN conceived the study, reviewed and critically appraised the selected papers, drafted the manuscript. YD performed the searching, reviewed and critically appraised the selected papers, reviewed the manuscript. KAG participated in drafting the manuscript and co-ordinated the project. All authors read and approved the final manuscript.

## Pre-publication history

The pre-publication history for this paper can be accessed here:



## Supplementary Material

Additional File 1 Search terms and strategies Click here for file

Additional File 2 Verification of study eligibility (sample) Click here for file

Additional File 3 Levels of evidence for assessing intervention studies (NHMRC 1999) Click here for file

Additional File 4 Summary of critical appraisal – Randomised controlled trials Click here for file

Additional File 5 Summary of critical appraisal – Non-randomised controlled trials Click here for file
