# Supplementary material for: Pelvic floor muscle training and adjunctive therapies for the treatment of stress urinary incontinence in women: a systematic review
Source: BMC Womens Health. 2006 Jun 28;6:11. doi: 10.1186/1472-6874-6-11 (PMC1586224; doi:10.1186/1472-6874-6-11)
Supplement: Additional File 1 — Search terms and strategies [file 1472-6874-6-11-S1.doc]

## Additional file 1: Search terms and strategies

The following search strategy was designed for MEDLINE but was also applied similarly in the other databases listed. All studies retrieved with a combination of keyword 1 with keyword 2 were reviewed regarding their title, abstract and descriptive terms for meeting the inclusion criteria.

Key words representing the intervention are listed under Keyword 1 (below). Key words representing impairments and disabilities are listed under Keyword 2.

***Keyword 1* *Keyword 2***

- Physiotherapy stress incontinence
- Physical therapy stress urinary incontinence
- Conservative management urinary stress incontinence
- Conservative therapy urinary incontinence
- Pelvic floor muscle training mixed incontinence
- Pelvic floor muscle exercises urodynamic stress incontinence
- Pelvic floor training genuine stress incontinence
- Electrotherapy
- Electrical stimulation
- Neuromuscular stimulation
- Biofeedback
- Cones
- Vaginal Cones
- EMG biofeedback
- Pressure biofeedback
- Vaginal pressure biofeedback
- Perineal biofeedback
- Bladder training
- Non-surgical treatment
- Non- pharmacological treatment
- Behavioral modification
- Myofeedback
