# Supplementary material for: Pelvic floor muscle training and adjunctive therapies for the treatment of stress urinary incontinence in women: a systematic review
Source: BMC Womens Health. 2006 Jun 28;6:11. doi: 10.1186/1472-6874-6-11 (PMC1586224; doi:10.1186/1472-6874-6-11)
Supplement: Additional File 2 — Verification of study eligibility (sample) [file 1472-6874-6-11-S2.doc]

## Additional file 2: Verification of study eligibility (sample)

| CITATION | | | | | TYPE OF STUDIES | | | PARTICIPANTS | | | OUTCOMES | |
| --- | --- | --- | --- | --- | --- | --- | --- | --- | --- | --- | --- | --- |
| Authors | Publication date | Title | Journal | Vol (issue):  pages | RCT/  non-RCT | Published 1995-2005 | English | Adult women | SUI | PFMT  +/-Physical Therapy | SUI | Clinical OM |
| Text | (Year) | Text | Text | Numbers | Yes/No | Yes/No | Yes/No | Yes/No | Yes/No | Yes/No | Yes/No | Yes/No |
|  |  |  |  |  |  |  |  |  |  |  |  |  |
|  |  |  |  |  |  |  |  |  |  |  |  |  |
|  |  |  |  |  |  |  |  |  |  |  |  |  |
|  |  |  |  |  |  |  |  |  |  |  |  |  |
|  |  |  |  |  |  |  |  |  |  |  |  |  |

RCT = randomised controlled trial, non-RCT = non-randomised controlled trial, SUI = stress urinary incontinence, PFMT = pelvic floor muscle training, OM = outcome measure
