# Supplementary material for: Pelvic floor muscle training and adjunctive therapies for the treatment of stress urinary incontinence in women: a systematic review
Source: BMC Womens Health. 2006 Jun 28;6:11. doi: 10.1186/1472-6874-6-11 (PMC1586224; doi:10.1186/1472-6874-6-11)
Supplement: Additional File 3 — Levels of evidence for assessing intervention studies (NHMRC 1999) [file 1472-6874-6-11-S3.doc]

## Additional file 3: Levels of evidence for assessing intervention studies (NHMRC 1999)

| I | Evidence obtained from a systematic review of all relevant randomised controlled trials |
| --- | --- |
| II | Evidence obtained from at least one properly-designed randomised controlled trial |
| III-1 | Evidence obtained from well-designed pseudorandomised controlled trials (alternate allocation or some other method) |
| III-2 | Evidence obtained from comparative studies (including systematic reviews of such studies) with concurrent controls and allocation not randomised, cohort studies, case-control studies, or interrupted time series with a control group |
| III-3 | Evidence obtained from comparative studies with historical control, two or more single arm studies, or interrupted time series without a parallel control group |
| IV | Evidence obtained from case series, either post-test or pre-test/post-test |
