# Supplementary material for: Pelvic floor muscle training and adjunctive therapies for the treatment of stress urinary incontinence in women: a systematic review
Source: BMC Womens Health. 2006 Jun 28;6:11. doi: 10.1186/1472-6874-6-11 (PMC1586224; doi:10.1186/1472-6874-6-11)
Supplement: Additional File 4 — Summary of critical appraisal – Randomised controlled trials [file 1472-6874-6-11-S4.doc]

## Additional file 4: Summary of critical appraisal – Randomised controlled trials

| Citation | Aksac 2003 | Arvonen 2001 | Berghmans 1996 | Bidmead 2004 | Bo 1999 | Cammu & van Nylen 1998 | Dumoulin 2004 | Bo 2000 | Glavind 1996 | Hay-Smith 2002 | Johnson 2001 | Knight 1998 | Miller 1998 | Morkved 2002 | Pages 2001 | Pieber 1995 | Wong 2001 | Total |
| --- | --- | --- | --- | --- | --- | --- | --- | --- | --- | --- | --- | --- | --- | --- | --- | --- | --- | --- |
| Study purpose clearly stated | 1 | 1 | 1 | 1 | 1 | 1 | 1 | 1 | 1 | 1 | 1 | 1 | 1 | 1 | 1 | 1 | 1 | 16 |
| Ethical processes | 1 | 1 | 1 | 1 | 1 | 0 | 1 | 1 | 1 | 0 | 1 | 1 | 1 | 1 | 1 | 0 | 1 | 13 |
| Lit. review relevant | 1 | 1 | 1 | 1 | 1 | 1 | 1 | 1 | 1 | 1 | 1 | 1 | 1 | 1 | 1 | 1 | 1 | 16 |
| Random allocation | 1 | 1 | 1 | 1 | 1 | 1 | 1 | 1 | 1 | 1 | 1 | 1 | 1 | 1 | 1 | 1 | 1 | 16 |
| Allocation concealed | 1 | 0 | 1 | 0 | 1 | 1 | 1 | 1 | 1 | 1 | 0 | 1 | 0 | 1 | 0 | 0 | 0 | 9 |
| Assessors blinded | 0 | 0 | 1 | 0 | 1 | 0 | 1 | 0 | 0 | 1 | 0 | 1 | 0 | 1 | 0 | 0 | 0 | 5 |
| Baseline similarity | 1 | 1 | 1 | 1 | 1 | 1 | 1 | 1 | 1 | 1 | 1 | 1 | 1 | 1 | 0 | 1 | 1 | 15 |
| Pre-stratification | 0 | 0 | 1 | 0 | 1 | 0 | 1 | 1 | 0 | 0 | 0 | 0 | 0 | 1 | 0 | 0 | 0 | 5 |
| Consideration of sample size | 0 | 0 | 0 | 0 | 1 | 0 | 1 | 1 | 0 | 1 | 1 | 0 | 0 | 1 | 0 | 0 | 0 | 6 |
| Inclusion/exclusion criteria | 0 | 1 | 1 | 0 | 1 | 1 | 1 | 1 | 1 | 1 | 1 | 1 | 1 | 1 | 1 | 1 | 1 | 14 |
| OM clearly described in Intro/Methods | 0 | 1 | 1 | 0 | 1 | 1 | 1 | 1 | 0 | 0 | 1 | 1 | 1 | 1 | 1 | 1 | 1 | 12 |
| OM reliable stated | 0 | 1 | 1 | 0 | 1 | 0 | 1 | 1 | 0 | 0 | 1 | 1 | 1 | 1 | 0 | 0 | 0 | 8 |
| OM valid stated | 0 | 1 | 1 | 0 | 1 | 0 | 1 | 1 | 0 | 0 | 0 | 1 | 1 | 1 | 0 | 0 | 1 | 8 |
| Intervention described to allow replication | 0 | 1 | 1 | 0 | 1 | 1 | 0 | 1 | 0 | 0 | 1 | 1 | 1 | 1 | 0 | 1 | 1 | 10 |
| Contamination avoided | 0 | 0 | 0 | 0 | 1 | 0 | 1 | 1 | 0 | 1 | 0 | 0 | 0 | 1 | 0 | 0 | 0 | 5 |
| Co-intervention avoided | 0 | 0 | 0 | 0 | 1 | 0 | 0 | 1 | 0 | 1 | 0 | 0 | 0 | 1 | 0 | 0 | 0 | 4 |
| Results reported in terms of significance | 1 | 1 | 1 | 1 | 1 | 1 | 1 | 1 | 1 | 1 | 1 | 1 | 1 | 1 | 1 | 0 | 1 | 15 |
| Analysis appropriate | 1 | 1 | 1 | 1 | 1 | 1 | 1 | 1 | 1 | 0 | 1 | 1 | 1 | 1 | 1 | 1 | 1 | 15 |
| Withdrawals/ dropouts reported | 0 | 1 | 1 | 1 | 1 | 1 | 1 | 1 | 1 | 1 | 1 | 1 | 1 | 1 | 1 | 1 | 1 | 15 |
| Intention to treat or no dropouts reported | 0 | 0 | 1 | 0 | 1 | 1 | 1 | 0 | 0 | 0 | 0 | 1 | 1 | 1 | 1 | 1 | 1 | 9 |
| Conclusions appropriate | 1 | 1 | 1 | 1 | 1 | 1 | 1 | 1 | 1 | 1 | 1 | 0 | 1 | 1 | 1 | 1 | 1 | 16 |
| Clinical importance reported | 0 | 0 | 1 | 1 | 1 | 1 | 1 | 1 | 1 | 1 | 1 | 1 | 1 | 1 | 1 | 1 | 1 | 14 |
| Limitations reported | 0 | 1 | 1 | 0 | 1 | 1 | 1 | 1 | 1 | 1 | 0 | 0 | 1 | 0 | 1 | 1 | 1 | 12 |
| Total | 9 | 15 | 20 | 10 | 23 | 15 | 21 | 21 | 13 | 15 | 15 | 17 | 17 | 22 | 13 | 13 | 16 |  |

## KEY: OM = Outcome Measure
