# Supplementary material for: Pelvic floor muscle training and adjunctive therapies for the treatment of stress urinary incontinence in women: a systematic review
Source: BMC Womens Health. 2006 Jun 28;6:11. doi: 10.1186/1472-6874-6-11 (PMC1586224; doi:10.1186/1472-6874-6-11)
Supplement: Additional File 5 — Summary of critical appraisal – Non-randomised controlled trials [file 1472-6874-6-11-S5.doc]

## Additional file 5: Summary of critical appraisal – Non-randomised controlled trials

| Citation | Chen 1999 | Dumoulin 1995 | Balmforth 2004 | Turkan 2005 | Parkkinen 2004 | Sung 2000 | Finkenhagen 1998 | Total |
| --- | --- | --- | --- | --- | --- | --- | --- | --- |
| Study purpose clearly stated | 1 | 1 | 1 | 1 | 1 | 1 | 1 | 7 |
| Ethical processes | 1 | 1 | 1 | 1 | 1 | 1 | 0 | 6 |
| Literature review relevant | 1 | 1 | 0 | 1 | 1 | 1 | 0 | 5 |
| Assessor blinded | 0 | 1 | 0 | 0 | 1 | 0 | 0 | 2 |
| Compliance reported | 1 | 0 | 0 | 0 | 1 | 0 | 0 | 2 |
| No biases present | 0 | 0 | 0 | 0 | 0 | 0 | 0 | 0 |
| Consideration of sample size | 0 | 0 | 0 | 0 | 0 | 0 | 0 | 0 |
| Inclusion/exclusion criteria | 1 | 1 | 0 | 1 | 0 | 0 | 0 | 3 |
| OM clearly described in Intro/methods | 0 | 1 | 0 | 0 | 1 | 1 | 1 | 4 |
| OM reliability stated | 0 | 0 | 0 | 0 | 0 | 0 | 0 | 0 |
| OM validity stated | 0 | 0 | 0 | 0 | 0 | 0 | 0 | 0 |
| Intervention described (to allow replication) | 0 | 1 | 0 | 1 | 1 | 0 | 1 | 4 |
| Results reported in terms of significance | 1 | 1 | 1 | 1 | 1 | 1 | 0 | 6 |
| Analysis appropriate | 1 | 1 | 1 | 1 | 1 | 1 | 0 | 6 |
| Withdrawals/ dropouts reported | 1 | 1 | 0 | 1 | 1 | 0 | 0 | 4 |
| Intention to treat, or no dropouts | 1 | 0 | 0 | 1 | 0 | 0 | 0 | 2 |
| Conclusions appropriate | 1 | 1 | 1 | 1 | 1 | 1 | 1 | 6 |
| Clinical importance reported | 1 | 1 | 1 | 1 | 1 | 1 | 1 | 6 |
| Limitations reported | 0 | 1 | 0 | 0 | 1 | 0 | 0 | 2 |
| Total | 11 | 13 | 6 | 11 | 13 | 8 | 5 |  |

## OM = Outcome Measure
